# Assessment and State of Nutrition of Patients with Gastroenteropancreatic Neuroendocrine Neoplasms

**DOI:** 10.3390/nu12071961

**Published:** 2020-06-30

**Authors:** Justyna Kikut, Anna Jasińska, Jakub Pobłocki, Jacek Brodowski, Szczuko Małgorzata

**Affiliations:** 1 Department of Human Nutrition and Metabolomics, Pomeranian Medical University in Szczecin, Broniewskiego 24, 71-460 Szczecin, Poland; justyna.kikut@pum.edu.pl (J.K.); a.jas@wp.pl (A.J.); 2 Department of Endocrinology, Metabolic Diseases and Internal Diseases, Pomeranian Medical University in Szczecin, Unii Lubelskiej 1, 70-252 Szczecin, Poland; jakub.poblocki@pum.edu.pl; 3 Primary Care Department, Pomeranian Medical University in Szczecin, Żołnierska 48, 71-210 Szczecin, Poland; jacek.brodowski@pum.edu.pl

**Keywords:** neuroendocrine neoplasms, gastroenteropancreatic neuroendocrine neoplasms, nutrition, methods of nutrition, nutritional status

## Abstract

Introduction: In recent decades, the number of gastro-entero-pancreatic neuroendocrine neoplasms (GEP-NENs) cases, associated with coexisting metabolic disorders, has been continuously increasing. Patients with progressing neoplastic disease are at a risk of malnutrition. To improve the quality of life of neuroendocrine neoplasms (NEN) patients, the therapeutic approach should be supported by a well-balanced diet. The aim of the study was to analyze the nutritional errors and deficits in a group of GEP-NET patients. Materials and methods: The study group included 26 GEP-NET patients; 13 men and 13 women. The mean age of women was 68.77 ± 8.0, and the mean age of men was 64.69 ± 8.1. Three interviews on consumption in the last 24 h were performed, in order to evaluate the quality and quantity of nutrition. The data was incorporated into a dietetics software, which allows one to calculate the number of over 58 micronutrients and macronutrients with the participation of 52 menus. Subsequently, the mean values were compared with the current nutritional standards. Results: An energy deficit was observed in the group of women—76.9%, and men—100%, as well as high fat consumption in 23.1% in both groups. The proportions of SFA/MUFA/PUFA were very negative, whereas the consumption of saccharose was too high. Vitamin D deficiency was observed in 100% of men and women. Moreover, both men and women experienced the deficiency of vitamin E, folates and niacin. The consumption of sodium and phosphorus was twice as high as recommended, and an insufficient supply of calcium was observed in 80% of women and 90% of men. The insufficient consumption of magnesium, iodine and potassium in a significant part of the studied group was observed. All participants consumed too much cholesterol and insufficient amounts of fiber. The healthy diet indicator (HDI) and diet quality index (DQI) scores were 3.1 ± 1.8 (HDI) and 3.7 ± 1.6 (DQI) for women, and 7.2 ± 2.6 (HDI) and 8.5 ± 2.4 (DQI) for men. Conclusions: When analyzing the nutrition of GEP-NET patients, we highlight that they do not have a proper diet, despite the fact that they changed the way they eat. Dietetics support and the development of official nutritional standards seem to be a necessary element in the therapy of GEP-NET patients.

## 1. Introduction

Neuroendocrine neoplasms (NENs) may occur in different body locations, and their classification results from the place of origin and hormonal activity [1]. Some of the NENs have the ability to produce and store various peptide hormones, such as: insulin, gastrin, vasoactive intestinal peptide, glucagon and somatostatin. NENs form a group of neoplasms that may originate:

- from neuroendocrine cells localized in different endocrine glands, such as the pancreas, thyroid, parathyroid glands, pituitary gland and adrenal gland
- from dispersed neuroendocrine cells, predominantly of the digestive and respiratory tract [1].

In 2010, they were classified by WHO as malignant neoplasms [2]. NENs are divided into two categories: well-differentiated neoplasms, i.e., neuroendocrine tumors (NETs), and poorly-differentiated NENs called neuroendocrine cancers (NECs) [3]. In recent decades, the number of gastro-entero-pancreatic neuroendocrine tumors (GEP-NETs) cases has been increasing, but it seems to be associated with the progress in diagnostics. The most frequent primary locations are the gastro-entero-pancreatic tract and the lung (respectively 62–67% and 22–27%) [4]. GEP-NETs are the second most frequent type of neoplasm of the gastrointestinal tract, just after colorectal cancer [5]. NETs present a heterogeneous group of malignancies; very diverse in terms of their clinical and biological properties [3]. The functional gastro-entero-pancreatic NETs (hormonally active GEP-NETs) include small bowel NETs (ileum and jejunum), which can produce serotonin, and maybe accompanied by a carcinoid syndrome. Functional pancreatic neuroendocrine tumors (f-pNETs) are able to produce gastrin, insulin, intestinal polypeptides and glucagon, and can lead to the occurrence of such clinical syndromes as ulcers, hypoglycemia, hyperglycemia and diarrhea [6]. Rare p-NETs like glucagonoma and VIPoma are associated with hypokalemia and watery diarrhea, but less frequently [3]. Otherwise, non-functional pNETs (nf-pNETs) can still secrete some amounts of peptides or amines, but the levels are so low that they do not cause symptoms or are metabolically inactive [3].

Excessive production of stomach-intestinal hormones, peptides and amines in the neoplasms of the gastrointestinal tract contributes to the intestinal peristalsis disorders and malabsorption, with frequently accompanying diarrhea [7].

The NET patient group includes people with metabolic disorders, which often lead to the development of diabetes and obesity, whose treatment is also based on the reduction of excessive fat mass. However, not following a reduction diet correctly may inhibit the treatment of the illness. This is why we analyzed the nutrition and supply of nutrients of our patients. Patients with progressing neoplastic disease are often at the risk of qualitative malnutrition associated with the insufficient consumption of deficit nutrients. In GEP-NET patients, malnutrition may be caused by, e.g., mesenteric tumor infiltration, the disorder of the function of hormones that influence gut transit, or by pharmacological treatment [8]. Moreover, this type of neoplasm often features diarrhea caused by excessive motility of the small intestine, stimulated by the excessive secretion of serotonin precursors [8]. For this reason, GEP-NET patients may present significant problems with the incorrect absorption of nutrients, vitamin deficiency or food intolerance [9]. GEP-NET patients are the group of people that are at the highest risk, therefore, proper nutrition is important for the proper support of treatment and improvement of the patients’ quality of life.

## 2. Materials and Methods

The study was conducted after receiving the permission of the Bioethics Committee of the Pomeranian Medical University (regulation no. KB-0012/116/15).

### 2.1. Study Group

The study group consisted of 26 patients treated due to GEP-NETs in the Department of Endocrinology, Metabolic Diseases and Internal Diseases, Pomeranian Medical University, Szczecin. In the investigation, 13 men and 13 women with diagnosed GEP-NETs participated.

The mean age of patients with neuroendocrine tumors was 68.8 ± 8 for women and 64.7 ± 8.1 for men. The other parameters of both groups of patients are presented in Table 1. The control group (CG) consisted of 20 potentially healthy people (10 men and 10 women). The anthropometric characteristics are presented below in Table 1.

The primary tumor location mainly concerned the pancreas in 6 women and 7 men, and the small intestine in 7 women and 6 men. In 21 GEP-NET patients (80.8%) metastasis was observed. The main sites of metastasis were the liver (69%) and the pancreas, lymph nodes and bones (about 10% each). Furthermore, 61.5% of our patients, at an earlier stage of the disease, underwent primary tumor resection. Moreover, the mean loss of body mass since the onset of the illness in this group of patients (on average: 4 years ago) was 8.46 ± 2.46 kg in women and 9.53 ± 3.28 kg in men. All patients with neuroendocrine tumors of the gastrointestinal tract were undergoing somatostatin analogues injections therapy.

### 2.2. Anthropometric Measurements

In order to evaluate the differences in the state of nutrition, the following anthropometric measurements were performed: body mass (±0.1 kg) and height (±0.5 cm), using medical scales (Radwag WPT 60/150 OW, Poland) with a stadiometer, as well as waist and hip circumference (±0.5 cm). On the basis of these parameters, BMI and WHR were calculated. Both BMI and WHR were compared to WHO norms [10]. Additionally, in the case of GEP-NETs, the percentage weight loss since the diagnosis of the onset of the illness (an average of 4 years) was also determined. WHO’s recommendations for waist circumference for obesity prevention in the European population are <80 cm for women and <94 cm for men. In terms of waist-hip ratio, calculated on the basis of waist and hip circumference, the recommended values are <0.85 cm for women and <0.9 cm for men, in order to prevent the development of metabolic disorders [10].

### 2.3. Diet Content Analysis

A 24 h consumption interview was performed 3 times to evaluate the nutrition of patients. The interviews featured 2 weekdays and 1 weekend day of every patient. An album of products and meal photos was used to ensure the collection of reliable information regarding the size of the consumed meals [11]. The interview was conducted by qualified dieticians. The menus were analyzed using qualitative and quantitative methods. In the qualitative analysis, we used tests for the evaluation of the quality of nutritional methods: the healthy diet indicator (HDI) and the diet quality index (DQI) [12]. The maximum number of points to achieve was 16 for DQI—the lower the number, the higher the quality of the menu. For the HDI test, the maximum number of points to acquire was 9, and the higher the number, the better the quality of the menu [12].

The Dieta 6D program, recommended by the National Centre of Nutritional Education (Warsaw, Poland), was used for quantitative analysis. The data acquired from the program include a group of 58 micronutrients and macronutrients that features, e.g., carbohydrates, fats, fatty acids, proteins of animal or plant origin together with 18 amino acids, 10 mineral constituents, 10 vitamins, cholesterol, fiber and the calorific value of the diet. The acquired data were compared with the current Nutritional Norms for the Polish Population [13].

### 2.4. The Comparison with Nutritional Norms

To interpret the results, we used norms in accordance with the mean age of the studied group, separately for both genders. We referred to the estimated average requirement (EAR) of the group or, when there was no such estimate, to the adequate intake (AI), as presented in Table 2. The evaluation of consumption with the EAR cut-off point is presented in Table 2A. In Table 2B, the results are compared to the level of AI. Table 2C shows the comparison between diet nutrients and nutritional norms. The levels of reference to norms were calculated with a ±10% margin.

Considering the mean age of the studied population as a reference point for nutritional norms, the age range was established at 66–75 years old for women and 51–65 for men. In reference to the norms of consumed energy, we used the average age range and the average body mass of both genders, as well as the average activity of the group at the level of PAL = 1.4. Similarly, the norm range for protein consumption was established on the basis of mean age and mean body mass.

### 2.5. Statistical Analysis

Statistica 13.3 (Statsoft, Kraków, Poland) was used in the statistical analysis of the acquired data. Because most of the data showed normal distribution (Shapiro–Wilk test), the analysis was conducted using a *t*-student test. Statistical significance was established at *p* ≤ 0.05.

## 3. Results

The study group consisted of 26 patients: 13 women and 13 men. The mean WHR of patients was 0.9 ± 0.1 for women and 1.0 ± 0.1 for men (Table 3). BMI values suggest that men were slightly overweight, at 25.5 ± 4.3, which is acceptable in older people. Considering waist and hip circumference, we observed visceral abdominal fat in both sexes.

On the basis of the acquired data, we conducted the evaluation of the diet in terms of quality, using the diet quality index (DQI) and the healthy diet indicator (HDI). The mean DQI was 7.2 ± 2.6 for women and 8.5 ± 2.4 for men. The maximum number of points to acquire was 16, where 16 meant a low quality diet. The acquired results classify the patients’ diets as low quality. This situation mainly resulted from excessive amounts of saturated fats and sodium, as well as insufficient amounts of calcium and leguminous vegetables in the diet. None of the respondents achieved the maximum number of points in the HDI test. The mean values were 3.1 ± 1.8 and 3.7 ± 1.6, respectively. The patients’ diets featured incorrect consumption of fats, dietary fiber, fruit and vegetables, leguminous plants, grains, nuts and excessive amounts of cholesterol (Table 3).

### 3.1. The Quantitative Analysis of the Menus

Table 4A–C and Table 5, Table 6 and Table 7 show the percentage or average consumption of specific nutrients for men and women, with reference to nutritional norms. Such an analysis makes it possible to observe the excess or deficit of nutrients. It is important to highlight that none of the nutrients’ single intake values exceeded the upper level (UL) to nutrient recommendations [13].

### 3.2. Nutrients

The energy deficit was 76.9% in the group of women and 100% in the group of men (Table 4B). The difference was statistically significant between both groups—some of the patients could have applied the diet merely under their basal metabolic rate. Protein consumption was at appropriate levels in both groups. In both women and man, the relation between animal and plant protein consumption was 2.6:1 (Table 4). In terms of the percentage of fat present in the diets, it exceeds the recommended norms in 23.1% of women and in 23.1% of men. At the same time, in 15.4% of men, the consumption of this constituent in the diet is insufficient. This situation was not observed in women (Table 4B). Cholesterol consumption in women did not exceed the norm, contrary to men. The amount of saturated fatty acids in both groups significantly exceeded the norm. The percentage of the consumption was 46.2% for both groups (Table 4B and Table 5).

In the case of carbohydrates, the entire study group followed the appropriate, mean consumption of carbohydrates in general, as well as the mean percentage participation in the diet (Table 5). However, an inadequate consumption of fiber was observed in both groups. The percentage of women and men who did not meet the fiber requirements was 76.9%. An excessive consumption of saccharose was observed in 38.5% of women and 23.1% of men, despite the fact that the average value in the group was appropriate (Table 4B and Table 5).

### 3.3. Vitamins and Minerals

In the case of vitamins, we observed a statistically significant difference for the consumption of niacin and vitamin C between men and women (in both groups: NEN and CG; however, sex relationships in vitamin C intake were opposed). Neither sex group in NEN and CG reached the required levels consumption of vitamin E and folates. In both cases, vitamin D consumption was at an unacceptably low level, and its deficiency concerned 100% of the patient population (Table 4C and Table 6). Furthermore, in both groups, we observed correct average consumption for vitamin A, B1, B2, B3, B6 and B12. However, referring to the percentage of consumption in women, we were observed deficiency at niacin at 50% level and 30% for tiamine and vitamin B6 in group NEN. In men, the largest deficiencies were referring to thiamine (46.2%) (Table 4A).

When it comes to the content of individual minerals, the levels of sodium and phosphorous were significantly above the norm in both men and women. Both groups (CG) exceeded the recommended consumption of sodium (Table 7). Furthermore, in terms of the percentage of consumption, the intake was too high in 92.3% of both men and women (Table 4C). The situation is similar in terms of the consumption of phosphorus (Table 4A). We also observed a decrease in the consumption of calcium, potassium, magnesium and iodine (Table 7). Similarly to vitamin D, calcium deficiency was observed in over 80% of men and 90% of women (Table 4C). Magnesium consumption deficiency was observed in 92.3% of men and 53.8% of women. Deficiency iodine was observed in both groups at 46.2%. Interestingly, the mean level of iron and zinc was appropriate in both groups (Table 7), but 30.8% of women and 26.3% of men did not consume enough zinc, and we observed iron deficiency in 15.4% of women (Table 4A).

Comparing nutrient intake in the control group with the NEN group, the following were observed: reducing energy, fat and animal products consumption. At the same time, patients included slightly more fiber and fish (iodine) on the menu. However, these changes were not satisfactory, and adversely affected the intake of most vitamins and minerals, and limited the availability of amino acids.

## 4. Discussion

Proper nutrition is very important in the treatment of neoplastic patients, in order to develop proper defense mechanisms and to decrease the risk of malnutrition. The latter is, in itself, an important prognostic factor for NEN patients, but it is not a characteristic symptom. It has been demonstrated that malnutrition has a significant influence on the number of complications and the time of hospitalization, as well as the mortality rate [14]. It is estimated that malnutrition in this group of patients can reach the level of 21–25% [14]. Maasberg et al. proved that 25.1% of NEN patients were at the risk of malnutrition according to the SGA scale, whereas 21.7% of hospitalized patients were at a high risk of malnutrition according to the NRS scale [14]. Furthermore, Shatwell et al. demonstrated, using the MUST scale, that malnutrition concerns 14% of GEP-NET patients with outpatient treatment [8]. Although our patients did not experience malnutrition, the analysis of their nutritional methods suggest the possibility of occurrence of multiple nutritional deficiencies. When increasing body content, particularly fat content as a factor in the occurrence of neoplasms, it is important to highlight that, in this study, WHR was 0.9 for women and 1.0 for men, suggesting excessive accumulation of fat tissue in the abdominal area. This observation is confirmed by waist circumference: 93 cm for women and 101 cm for men. In terms of BMI, for patients aged >65, the reference values are 25–27, and these BMI values are associated with a lower risk of mortality [15]. Furthermore, it is also worth highlighting the presence of a neoplastic disease, during which this seemingly slight excess of body weight may have a protective effect against the occurrence of malnutrition. It would seem that patients with a neuroendocrine pancreatic tumor are at risk of increasing this index, as a significantly higher number of patients with the tumor had BMI > 30 [16]. Zhan et al. observed higher BMI in the study group, but the authors associate this situation with the presence of insulinoma in these patients [17]. In a different study, it was observed that the coexistence of obesity in patients significantly correlates with a decreased mortality rate and, in accordance with the conclusion, malnutrition was associated with increased mortality in NET patients [18]. Barrea et al. also observed higher BMIs, indicating overweight in the group of GEP-NET patients [19]. In a prospective study, Cross et al. observed a positive correlation between higher BMI and the presence of malicious tumors of the small intestine. This observation further highlights the important role of nutrition [20]. Gallo et al. achieved results that were similar to the ones presented in our study in terms of anthropometric measurements, observing that overweight was present in over 40% of participants, obesity in 28.1%, and mean waist circumference was determined at 95.1 ± 17.2 cm [7]. When analyzing accompanying illnesses, similarly to our study, a higher frequency of occurrence of diabetes was observed in NEN and hypertension cases [16]. Hassan et al. observed that, a year before the diagnosis of pNET, diabetes was observed [21].

The quantitative analysis of the studied menus indicated caloric deficits, which were most probably associated with decreased appetite and feeling full faster, due to the ongoing disease or therapy that aimed to reduce excess fat tissue. This is somewhat confirmed by a survey study in which the change of diet caused by diagnosed NET was reported by 58% of the respondents [22]. Furthermore, NET patients report lower quality of life in comparison to the control group [23]. In this study, patients did not experience protein deficiency. Both groups consumed increased amounts of animal protein. The ratio between animal and plant protein consumption was 2.6:1 in both groups. A similar 3:1 result was observed in the study by Barrea et al. [19]

None of the patients consumed enough dietary fiber. The mean intake of this constituent was only 13 g in women and 15 g in men. The results indicate insufficient intake of fiber in the form of vegetables, fruit or grain products. This fact is also confirmed by the qualitative analyses of menus, which confirmed deficiencies in the menus in terms of legumes, wholegrain products, fruit and vegetables, both raw and processed. Similar values were achieved in a study by Gallo et al. [7] The exacerbating symptoms in the gastrointestinal tract had an influence on the limitation of fiber consumption, further contributing to the presence of deficiencies of antioxidants, as well as to changes in gut microflora [9,24].

The analysis of the menus revealed critically low vitamin D levels in both groups, with reference to the average and percentage of consumption. A study of patients with neuroendocrine tumors of the small intestine revealed critically low levels of vitamin D in the blood of as many as 29% of patients, and 17% of people experienced moderate deficiency [25]. Similarly, a different study indicated an extreme deficiency of vitamin D, under 10 ng/mL in 33% of patients, whereas 68% of the studied population experienced levels under 20 ng/mL [26]. In this study, we did not measure the state of vitamin D in blood serum, but due to the fact that its concentration correlates with the stage of illness as well as the ongoing therapy, a biochemical deficiency should be expected as well. In some patients with neuroendocrine neoplasms, there is only limited absorption of vitamin D, due to the treatment with somatostatin analogues [27], which were applied in the case of all our NEN patients. In terms of vitamins soluble in fats—A, D, E, K—78% of patients experienced the deficiency of one of these constituents [28]. In this study, we also observed the insufficient intake of these vitamins with the diet. The level of vitamin E in the erythrocytes of patients oscillated at the level of 58% in a study by Fiebrich et al. [28] Furthermore, it is suggested to supplement vitamins soluble in fats, particularly when there are problems with absorption and fatty diarrhea [29]. Interestingly, niacin deficiencies were not observed in the studied group with the mean consumption level. However, if we focus on the percentage of consumption, the deficiency was observed in 53.8% of women and in 23.1% of men. In terms of the biochemical deficiency of niacin, it was observed that the deficiency significantly appeared more often in the group of patients with a carcinoid than in the control group [30]. It was also suggested that the deficiency of this vitamin may serve as a biochemical prognostic marker for the identification of patients with a carcinoid [31]. Insufficient consumption of this vitamin may be a result of the increased metabolism of tryptophan into serotonin, and it may lead to diarrhea, dementia or even death [29]. Critically low dietary folate intake was observed in this study. Their deficiency affected 84.6% of men and as much as 92.3% of women. Low folate concentration interferes with DNA synthesis by limiting methylation and the ability to repair damaged chromosomes, which stimulates the development of brain, lung, pancreatic, ovarian, cervical and intestinal cancers [32].

With reference to minerals, their content in the diet of this group of patients was not studied in detail in the subject literature. However, in cancer patients, selenium and zinc deficiencies can have an influence on, e.g., the weakening of the immune response [33]. In this study, we observed insufficient consumption, especially of calcium, potassium, magnesium and iodine. Zinc deficiency was not observed in the mean consumption of the group, but such deficiencies were observed when analyzing the percentage of consumption in over 30% of the studied patients.

An excessive consumption of phosphorus and sodium was observed. The intake exceeded the norm twice. As studies on mice revealed that the cell cycle is affected by kinase B, which facilitates organic phosphorus cascade in the cytoplasm and nucleus—accelerating cell cycle and neoplasm formation, the high intake of phosphorus may have an enormous impact on the health of patients [34,35]. This is why there should be a strict ban on the consumption of smoked meat rich in phosphates. It is important to direct the patients’ attention to products rich in this constituent, as its excessive intake is associated with a higher risk of death [34]. The main recommendations for this group of patients were formulated by Warner et al. at Carcinoid Cancer Foundation. A high protein diet is recommended: 1–1.5 g/kg. This is particularly important due to the frequent deficiencies of niacin, which is created from the tryptophan that occurs in proteins. The recommendations also focus on the limitation of the consumption of fat to 30%, especially in people with a carcinoid and a tendency to have diarrhea [36]. However, it is highlighted that a diet’s constituents should be individually adjusted to the needs of the patient [37]. Moreover, NEN patients with hypersensitivity should be wary of certain products that feature biogenic amines [37]. Products that they should avoid include, e.g., ripe cheese, smoked meat, alcohol—wine/beer, soy products, broad bean, sauerkraut, tomatoes, avocados, bananas, raspberries, pineapples and chocolate [33]. Other dietary recommendations may refer to frequently consumed medicines metabolized by P450 cytochrome—CYP3A4. This is why those that are ill should avoid food that may influence the cytochrome, changing the metabolism of the medicine. CYP3A4 inhibitors include, e.g., garlic, cranberry, resveratrol and soy [38,39]. Furthermore, some studies indicate that if the Mediterranean diet’s recommendations are not followed properly, there may be an exacerbation of NET [7,19].

NET patients whose excessive hormone production caused symptoms of the gastrointestinal tract, such as diarrhea or vomiting (which may lead to the malabsorption of nutrients), should receive dietary recommendations, taking into account supplementation [7]. When analyzing the method of nutrition of NEN patients, it is important to highlight that patients nourish themselves incorrectly, despite the fact that their diets have changed. Proper diets support treatment and prevent further metastasis and the development of cachexia associated with the advanced stage of the illness. Dietetics support and the development of official nutritional standards seem to be necessary elements in the therapy of NEN patients.

A slight limitation in our study is the relatively small study group, therefore we suggest conducting a study on a larger cohort of patients.

## 5. Conclusions

The largest deficiencies in both groups (women, men) concerned niacin, folates, vitamins D and E, and calcium. To prevent niacin deficiency, patients’ diets should be enriched with products such as fish, meat and legume seeds. If it is impossible to supplement the deficiency with nutrition, it is recommended to introduce supplementation [40,41]. In order to ensure an adequate supply of folates, green leafy vegetables and legumes should be included in the diet, and their dietary contribution is positively correlated with the level of folates in the plasma [42]. On the other hand, the supply of fruit and vegetables recommended by the American Cancer Society will ensure the right amount of antioxidant vitamins, such as vitamins E and C [43].

The diet in the group of patients with gastro-entero-pancreatic neuroendocrine neoplasms (GEP-NENs) significantly deviates from the principles of rational nutrition. The qualitative and quantitative analysis of patients’ menus showed numerous irregularities, despite the diet modification. A significant caloric deficit, different minerals and vitamin deficiencies have been reported. An insufficient intake of adequate calories, especially during cancer, may contribute to malnutrition. The introduction of nutritional education among patients with GEP-NENs is the right direction to support their oncological treatment. Due to the variety of symptoms, nutritional status and NEN-associated diseases, nutritional approaches for each patient should be individualized [37].

## Figures and Tables

**Table 1:** The characteristics of the studied gastro-entero-pancreatic neuroendocrine tumors (GEP-NETs) patients (NEN) and control group (CG).

Parameter	NEN		*p* Value
	Women Avg ± SD	Men Avg ± SD	
Age (years)	68.8 ± 8	64.7 ± 8.1	NS
Body mass (kg)	58.7 ± 10	79.3 ± 14.8	0.0004
Height (m)	1.6 ± 0.04	1.8 ± 0.1	0.0000
Waist circumference (cm)	93 ± 15	101.8 ± 12.7	NS
Hip circumference (cm)	99.3 ± 10	102.6 ± 4.4	NS
Hypertension	61.5%	38.5%	NS
Diabetes	53.8%	38.5%	NS
**Parameter**	**Control Group (CG)**		***p* Value**
	**Women Avg ± SD**	**Men Avg ± SD**	
Age (years)	62.3 ± 12.2	63.5 ± 10.1	NS
Body mass (kg)	65.8 ± 7.8	84.1 ± 14.6	0.0007
Height (m)	1.61 ± 0.06	1.79 ± 0.1	0.0000

NS—not statistically significant.

**Table 2:** The assumed nutritional norms.

**A.** Evaluation of nutrition with the use of estimated average requirement (EAR) cut-off point.		
**Parameter**	**EAR Norm Levels**	
	**Women**	**Men**
Protein (g)	33–55	40–62
P (mg)	580	580
Mg (mg)	265	350
Zn (mg)	6.8	9.4
Cu (mg)	0.7	0.7
Iodine (µg)	95	95
Vitamin A (µg)	530	630
Vitamin B1 (mg)	0.9	1.1
Vitamin B2 (mg)	0.9	1.1
Niacin (mg)	11	12
Vitamin B6 (mg)	1.3	1.4
Folates (µg)	520	320
Vitamin B12 (µg)	2.0	2.0
Vitamin C (mg)	70	75
**B.** Evaluation of nutrition using the adequate intake (AI) level.		
**Parameter**	**The Level of AI**	
	**Women**	**Men**
Na (mg)	1300	1400
K (mg)	3500	3500
Ca (mg)	1000	800
Fe (mg)	6	6
Manganese (mg)	1.8	2.3
Vitamin D (µg)	15	15
Vitamin E (mg)	10	10
**C.** Evaluation of nutrition with the use of the norm of nourishment.		
**Parameter**	**The Assumed Norm**	
	**Women**	**Men**
Energy (kcal)	1750	2300
Protein (%)	15–20	15–20
Fat (%)	20–35	20–35
NKT (g)	12–14.4	16.5–19.8
Cholesterol (mg)	<300	<300
Carbohydrates (%)	45–65	45–65
Saccharose (g)	25	25
Dietary fibre (g)	20	25

**Table 3:** The anthropometric indexes of GEP-NET patients with quality diet index with gender division.

Parameter	Women Mean ± SD	Men Mean ± SD	*p*
BMI (kg/m^2^)	23.8 ± 4	25.5 ± 4.3	NS
WHR	0.9 ± 0.1	1.0 ± 0.1	0.0140
PPM	1182.1 ± 112	1602.1 ± 230.3	0.0000
CPM	1556.5 ± 189	2142.7 ± 314.2	0.0000
DQI	7.2 ± 2.6	8.5 ± 2.4	NS
HDI	3.1 ± 1.8	3.7 ± 1.6	NS

**Table 4:** The percentage of men and women with reference to the norms [%].

**A.** The percentage of men and women with reference to the EAR norm.				
**Constituent**	**% of Women**		**% of Men**	
	**<EAR**	**>EAR**	**<EAR**	**>EAR**
Protein (g)	-	61.5	23.1	61.5
P (mg)	-	84.6	-	100
Mg (mg)	53.8	30.8	92.3	-
Zn (mg)	30.8	58.3	38.5	30.8
Cu (mg)	38.5	46.2	7.7	38.5
Iodine (µg)	46.2	23.1	46.2	38.5
Vitamin A (µg)	23.1	69.2	23.1	46.2
Vitamin B1 (mg)	30.8	38.5	46.2	30.8
Vitamin B2 (mg)	15.4	76.9	23.1	53.8
Niacin (mg)	53.8	38.5	23.1	61.5
Vitamin B6 (mg)	30.8	46.2	23.1	61.5
Folates (µg)	92.3	-	84.6	-
Vitamin B12 (µg)	23.1	76.9	5.3	73.7
Vitamin C (mg)	69.2	15.4	7.7	61.5
**B.** The percentage of men and women with reference to the AI norm.				
**Constituent**	**% of Women**		**% of Men**	
	**<AI**	**>AI**	**<AI**	**>AI**
Na (mg)	-	92.3	-	92.3
K (mg)	84.6	-	100	-
Ca (mg)	84.6	7.7	92.3	7.7
Fe (mg)	15.4	61.5	-	84.6
Manganese (mg)	23.1	69.2	30.8	46.2
Vitamin D (µg)	100	-	100	-
Vitamin E (mg)	92.3	7.7	92.3	-
**C.** The percentage of men and women with reference to nutritional norms.				
	**% of Women**		**% of Men**	
	**<norm**	**>norm**	**<norm**	**>norm**
Energy (kcal)	76.9	-	100	-
Protein (%)	-	30.8	-	38.5
Fat (%)	-	23.1	15.4	23.1
NKT (g)	-	46.2	-	46.2
Cholesterol (mg)	-	46.2	-	38.5
Carbohydrates (%)	7.7	-	7.7	-
Saccharose (g)	-	38.5	-	23.1
Dietary fibre (g)	76.9	-	76.9	-

**Table 5:** The comparison of mean nutrient consumption (proteins including amino acids, fats, carbohydrates).

Constituents	Women (NEN) Avg ± SD	Men (NEN) Avg ± SD	*p*
Energy (kcal)	1320 ± 407.7	1498.6 ± 347.5	NS
Total proteins (g)	65.4 ± 24.6	81.8 ± 26.6	0.0250
Animal protein (g)	47.5 ± 21.1	59.3 ± 24.7	NS
Plant protein (g)	17.9 ± 7	22.5 ± 6.2	0.0151
% energy from protein	18.9 ± 4.3	21.7 ± 3.6	NS
Isoleucine (mg)	3282.1 ± 1293.3	4110.4 ± 1449.6	0.0345
Leucine (mg)	5112.3 ± 1973.1	6261.6 ± 2066.3	0.0455
Lysine (mg)	4534.6 ± 1828.8	5651.7 ± 2233.6	NS
Methionine (mg)	1635.2 ± 663.5	2089.7 ± 738.9	0.0237
Cystine (mg)	962.5 ± 345.4	1242 ± 347.6	0.0054
Phenylalanine (mg)	2888.3 ± 1080.8	3589 ± 1071.2	0.0229
Tyrosine (mg)	2436.5 ± 971.8	3019.3 ± 1016.5	0.0396
Threonine (mg)	2695.9 ± 1042.6	3422.9 ± 1141.6	0.0203
Tryptophan (mg)	842.5 ± 340.4	1075.4 ± 388.1	0.0257
Valine (mg)	3825.2 ± 1455.4	4761.8 ± 1578.3	0.0307
Arginine (mg)	3284 ± 1324.6	3899.3 ± 1316	NS
Histidine (mg)	1885.7 ± 760.7	2422.8 ± 1005.3	0.0346
Alanine (mg)	3228.9 ± 1283.9	4205.6 ± 1559.3	0.0172
Aspartic acid (mg)	5631.9 ± 2110.5	7079.9 ± 2369.1	0.0240
Glutamic acid (mg)	12,515.3 ± 4544.3	15,559.5 ± 4840.3	0.0234
Glycine (mg)	2771.2 ± 1092.5	3680.1 ± 1390.6	0.0116
Proline (mg)	4646.6 ± 1844.2	5518.5 ± 1813.6	NS
Serine (mg)	3204 ± 1249.4	3950.8 ± 1188	0.0318
Fat (g)	49.9 ± 21.5	52.9 ± 23.5	NS
NKT (g)	19.8 ± 10.9	21 ± 10.3	NS
MUFA (g)	19.3 ± 8.7	21.6 ± 11.3	NS
PUFA (g)	6.8 ± 4	6.3 ± 2.3	NS
LCP (g)	0.4 ± 0.7	0.1 ± 0.1	0.0323
Cholesterol (mg)	270.6 ± 142.8	336.4 ± 162.6	NS
% energy from fat	33.2 ± 6.9	31.1 ± 10.5	NS
Total carbohydrates (g)	164.4 ± 51.7	186.9 ± 51.4	NS
Assimilable carbohydrates (g)	151 ± 48.8	171.8 ± 47.8	NS
Dietary fibre (g)	13.5 ± 5.4	15.1 ± 6.2	NS
Saccharose (g)	23.4 ± 16.6	17 ± 11.7	NS
Lactose (g)	12.1 ± 11	5.7 ± 6.5	0.0131
Starch (g)	94.2 ± 30.4	131.8 ± 41.3	0.0005
% energy from carbohydrates	46.7 ± 6.4	47.1 ± 9.5	NS
**Constituents**	**Women (CG) Avg ± SD**	**Men (CG) Avg ± SD**	***p***
Energy (kcal)	1695.5 ± 184.8	2055.4 ± 271.0	0.000
Total proteins (g)	78.3 ± 18.2	88.4 ± 20.0	0.018
Animal protein (g)	52.4 ± 19.7	60.6 ± 18.4	0.023
Plant protein (g)	25.8 ± 6.2	27.7 ± 7.4	0.151
% energy from protein	20.1 ± 5.3	18.9 ± 3.3	NS
Isoleucine (mg)	3651.9 ± 791.9	3853.4 ± 943.1	NS
Leucine (mg)	5812.5 ± 1473.1	5875.2 ± 1766.3	NS
Lysine (mg)	4710.5 ± 1222.8	5114.9 ± 1386.5	NS
Methionine (mg)	1814.6 ± 545.5	1923.5 ± 637.8	NS
Cystine (mg)	1129.5 ± 325.2	1280.5 ± 357.1	0.069
Phenylalanine (mg)	3276.0 ± 1178.8	3719.5 ± 1221.6	0.086
Tyrosine (mg)	2756.1 ± 865.8	3089.3 ± 1075.2	NS
Threonine (mg)	3146.8 ± 1042.6	3323.5 ± 1071.6	0.058
Tryptophan (mg)	964.9 ± 281.1	1043.8 ± 347.8	NS
Valine (mg)	4263.0 ± 1253.8	4761.8 ± 1383.5	0.031
Arginine (mg)	3892.6 ± 850.3	4238.6 ± 938.4	NS
Histidine (mg)	2387.4 ± 578.2	2084.0 ± 975.3	0.097
Alanine (mg)	3623.5 ± 792.3	3755.9 ± 992.3	NS
Aspartic acid (mg)	6674.1 ± 1267.8	6563.0 ± 1542.4	NS
Glutamic acid (mg)	15,027.5 ± 2598.9	15,880.3 ± 3290.8	NS
Glycine (mg)	3108.9 ± 884.4	3480.1 ± 1097.9	NS
Proline (mg)	5182.9 ± 1227.2	5552.4 ± 1316.7	NS
Serine (mg)	3658.3 ± 1249.4	3965.9 ± 1098	NS
Fat (g)	60.7 ± 18.5	86.8 ± 13.6	0.001
NKT (g)	24.1 ± 8.9	32.1 ± 10.9	0.031
MUFA (g)	23.3 ± 7.7	35.9 ± 8.3	0.000
PUFA (g)	8.9 ± 3.9	13.2 ± 6.3	0.080
LCP (g)	0.4 ± 0.7	0.1 ± 0.1	0.032
Cholesterol (mg)	270.1 ± 108.8	384.9 ± 168,5	0.059
% energy from fat	31.8 ± 4.9	37.4 ± 8.5	0.058
Total carbohydrates (g)	218.7 ± 24.3	257.9 ± 41.3	0.038
Assimilable carbohydrates (g)	198.6 ± 42.1	240.7 ± 23.5	0.011
Dietary fibre (g)	20.1 ± 6.4	17,5 ± 5.2	NS
Saccharose (g)	40.4 ± 13.6	27 ± 14.3	0.053
Lactose (g)	7.5 ± 6.7	5.4 ± 4.6	NS
Starch (g)	119.7 ± 21.4	167.8 ± 19.3	0.000
% energy from carbohydrates	47.9 ± 6.2	47.6 ± 10.5	NS

NKT—saturated fatty acids; MUFA—monounsaturated fatty acids; PUFA—polyunsaturated fatty acids; LCP—long chain polyunsaturated fatty acids; NS—not significant statistically.

**Table 6:** Vitamin consumption comparison.

Constituents	Women (NEN) Avg ± SD	Men (NEN) Avg ± SD	*p*
Vitamin A (µg)	718.6 ± 421.6	827.3 ± 522.4	NS
Retinol (µg)	285.5 ± 139	301.9 ± 174.2	NS
Beta carotene (µg)	2638.6 ± 2279.3	3171.4 ± 2861.3	NS
Vitamin E (mg)	5.2 ± 2.3	6.1 ± 2.3	NS
Thiamine (mg)	1 ± 0.4	1.2 ± 0.6	NS
Riboflavin (mg)	1.4 ± 0.6	1.3 ± 0.4	NS
Niacin (mg)	12.6 ± 7.6	18.4 ± 9.7	0.0200
Vitamin B6 (mg)	1.5 ± 0.6	1.7 ± 0.6	NS
Vitamin C (mg)	44.2 ± 26.2	65.7 ± 46.7	0.0458
Vitamin B12 (µg)	3.6 ± 2.7	2.8 ± 1.1	NS
Vitamin D (µg)	2.8 ± 2.8	1.8 ± 1.1	NS
Folates (µg)	190.4 ± 74	215.4 ± 61.2	NS
**Constituents**	**Women (CG) Avg ± SD**	**Men (CG) Avg ± SD**	***p***
Vitamin A (µg)	1044.1 ± 491.6	1001.3 ± 488.4	NS
Retinol (µg)	361.2 ± 163.1	692.7 ± 435.5	0.042
Beta carotene (µg)	4123.6 ± 2738.2	2157.4 ± 1967.3	0.067
Vitamin E (mg)	7.8 ± 2.8	9.7 ± 4.8	NS
Thiamine (mg)	1.2 ± 0.3	1.6 ± 0.4	0.041
Riboflavin (mg)	1.4 ± 0.3	1.5 ± 0.4	NS
Niacin (mg)	15.6 ± 4.6	16.4 ± 5.1	NS
Vitamin B6 (mg)	1.7 ± 0.5	1.7 ± 0.5	NS
Vitamin C (mg)	92.5 ± 45.5	70.9 ± 47.0	0.048
Vitamin B12 (µg)	3.2 ± 2.0	4.1 ± 2.0	NS
Vitamin D (µg)	2.7 ± 2.2	3.2 ± 1.8	NS
Folates (µg)	283.4 ± 72.2	224.4 ± 92.4	0.091

NS—not significant statistically.

**Table 7:** The comparison of mineral consumption.

Minerals	Women (NEN) Avg ± SD	Men (NEN) Avg ± SD	*p*
Sodium (mg)	2489.7 ± 887.5	2764.1 ± 935.4	NS
Potassium (mg)	2170.8 ± 850.8	2272 ± 675.3	NS
Calcium (mg)	566.8 ± 333.2	448.6 ± 269	NS
Phosphorus (mg)	1099.2 ± 396.5	1202.1 ± 373.4	NS
Magnesium (mg)	237.6 ± 101.7	233.7 ± 67.1	NS
Iron (mg)	7.5 ± 2.4	8.4 ± 1.9	NS
Zinc (mg)	8 ± 2.8	9.3 ± 2.5	NS
Copper (mg)	0.8 ± 0.4	0.8 ± 0.2	NS
Manganese (mg)	3 ± 1.6	3.3 ± 1.7	NS
Iodine (µg)	82.6 ± 35.5	80.7 ± 42.8	NS
**Minerals**	**Women (CG) Avg ± SD**	**Men (CG) Avg ± SD**	***p***
Sodium (mg)	3099.7 ± 931.2	3538.0 ± 813.4	NS
Potassium (mg)	2830.1 ± 622.2.8	2594.7 ± 656.9	NS
Calcium (mg)	569.8 ± 283.1	454.1 ± 276.5	NS
Phosphorus (mg)	1203.7 ± 296.5	1252.9 ± 321.1	NS
Magnesium (mg)	283.5 ± 80.5	261.1 ± 91.9	NS
Iron (mg)	10.3 ± 2.3	11.4 ± 2.0	NS
Zinc (mg)	9.7 ± 2.3	10.9 ± 2.4	NS
Copper (mg)	1.1 ± 0.3	1.0 ± 0.2	NS
Manganese (mg)	4.3 ± 2.8	3.5 ± 1.9	NS
Iodine (µg)	62.8 ± 26.4	67.2 ± 28.9	NS

NS—not significant statistically.
